# *In Vitro* Characterization of a Multifunctional Staphylokinase Variant with Reduced Reocclusion, Produced from Salt Inducible *E. coli* GJ1158

**DOI:** 10.1155/2013/297305

**Published:** 2013-08-13

**Authors:** K. K. Pulicherla, Anmol Kumar, G. S. Gadupudi, Seetha Ram Kotra, K. R. S. Sambasiva Rao

**Affiliations:** ^1^Centre for Bioseparation Technology, VIT University, Vellore 632014, Tamilnadu, India; ^2^Department of Biotechnology, Acharya Nagarjuna University, Nagarjuna Nagar, Guntur 522510, Andhra Pradesh, India; ^3^Interdisciplinary Graduate Program in Human Toxicology, University of Iowa, Iowa City, IA 52242, USA; ^4^Department of Occupational and Environmental Health, The University of Iowa, College of Public Health, 100 Oakdale Campus No. 121 IREH, Iowa City, IA 52242-5000, USA

## Abstract

The thrombolytic therapy with clinically approved drugs often ensues with recurrent thrombosis caused by thrombin-induced platelet aggregation from the clot debris. In order to minimize these problems, a staphylokinase (SAK)-based bacterial friendly multifunctional recombinant protein SRH (staphylokinase (SAK) linked with tripeptide RGD and dodecapeptide Hirulog (SRH)) was constructed to have Hirulog as an antithrombin agent and RGD (Arg-Gly-Asp) as an antiplatelet agent in the present study. This multifunctional fusion protein SRH was expressed in osmotically inducible *E. coli* GJ1158 as soluble form and purified with a yield of 0.27 g/L and functionally characterized *in vitro*. SRH retained the fibrinolytic activity and plasminogen activation rate comparable to the parental counterpart SAK. The antithrombin activity of SRH was significantly higher than SAK. The platelet rich clot lysis assay indicated that SRH had enhanced platelet binding activity and *T*_50%_ and C_50_ of SRH were significantly lower than that of SAK. Furthermore, SRH inhibited the ADP-induced platelet aggregation in dose-dependent manner while SAK had no significant effect on platelet aggregation. Thus, the current study suggests that the SAK variant produced from osmotically inducible GJ1158 is more potent thrombolytic agent with antithrombin and antiplatelet aggregation activities for reduction of reocclusion in thrombolytic therapy.

## 1. Introduction

Thrombotic disorders are one of the major causative factors for worldwide mortality and morbidity. Millions of deaths are reported every year due to thrombosis in the United States and European countries [1, 2]. Thrombi can be localized anywhere in the circulatory system which are usually associated with myocardial infarction, stroke, peripheral occlusive disease, deep vein thrombosis, and pulmonary embolism. Thrombolytic therapy is a well-established treatment for these disorders in which the fibrinolytic system of the patient is activated by infusing the plasminogen activators. Streptokinase, urokinase, tissue plasminogen activator (t-PA), and their derivatives are widely used as fibrinolytic agents. Although these drugs reduced the morality rate, reocclusion problem is associated with the treatment. The thrombin that is released during the thrombolysis of clot activates the coagulation system and promotes platelet degranulation which subsequently leads to reocclusion [3]. Further, the reoccluded clots are usually platelet rich and resistant to some thrombolytic agents such as t-PA due to presence of platelet plasminogen activator inhibitor-1 (PAI-1) released by platelets [4]. Although the widely used t-PA offers good clinical efficacy and higher survival rates compared to currently approved drugs, only a few patients are able to afford its much higher cost ($US1400 per person) [5]. Hence, the choice of more potent thrombolytic drugs, which provide a fast and complete reperfusion with minimal side effects at low cost, is needed.

Staphylokinase, a clot specific profibrinolytic agent secreted by certain lysogenic *Staphylococcus aureus* strains [6], is a plasminogen activator and promising blood clot dissolving agent. Staphylokinase forms a 1 : 1 stoichiometric complex with inactive human plasminogen (hPg) circulating in the blood [7] which is followed by cleavage of 10 amino acids from N-terminal by hydrolysis of Lys^10^-Lys^11^ peptide bond and finally forms catalytically active SAK [8]. This catalytically active SAK-plasminogen complex later forms a ternary complex with another molecule of plasminogen and converts this plasminogen to plasmin (hPm) after the cleavage of the Arg^561^-Val^562^ peptide bond in hPg. This resultant SAK-plasmin complex functions as a plasminogen activator to convert plasminogen to plasmin in clot lysis [9]. SAK-plasmin complex is rapidly neutralized by the circulatory plasmin inhibitor, *α*_2_-antiplasmin, when the complex is not fibrin bound. In contrast, the fibrin-bound plasminogen activator complex is much more resistant (approx. 100 fold) to *α*_2_-antiplasmin-mediated inhibition which enables the activation of plasminogen on the thrombus surface without the systemic fibrinolytic activation [10]. 

The activated platelets and the released thrombin from the site of vascular injury due to the thrombus dissolution are mainly responsible for formation of secondary clots that causes reocclusion. Surface glycoprotein GPIIb/IIIa on activated platelets binds with fibrinogen to form platelet aggregates [11]. So, to avoid platelet aggregation, Arg-Gly-Asp (RGD) peptide, a GPIIb/IIIa receptor antagonist was used as antiplatelet factor in the protein. A 20-amino-acid peptide, Hirulog (FPRPGGGGNGDFEEIPEEYL) has been used as a thrombin inhibitor in the fusion protein. RGD and Hirulog were fused with staphylokinase at its C-terminal end without disturbing the N-terminal. A six-amino-acid linker was used to link the SAK and Hirulog to prevent the interdomain interaction for functional sustainability of the fusion protein. 

The aim of this study is to construct a multifunctional recombinant staphylokinase variant SRH (SAK-RGD-Hirulog) with reduced reocclusion which can be produced at economical price. The osmotically inducible *E. coli* GJ1158 expression system was selected to overcome the problem of high cost and toxicity of IPTG (isopropyl-*β* D-thiogalactopyranoside) in BL21 (DE3) expression system [12, 13]. In the yeast expression system, in spite of their capability of growing to high cell density, there is a possibility of unwanted glycosylation which can be antigenic [14]. Another drawback with the use of yeast-based expression systems is the added time required for development and screening of different transformed cell lines with suitable expression levels due to reliability on genomic insertion of cDNA, which creates expression variability between the cell lines [15]. *E. coli* GJ1158 host containing a chromosomally integrated T7 RNA polymerase gene downstream of an osmotically inducible proUP promoter addresses most of the problems associated with protein expression in *E. coli* BL21 (DE3) and yeast system [13, 16]. 

Various SAK-based multifunctional recombinant proteins were constructed and expressed in different expression system in earlier studies to reduce the reocclusion and bleeding complications. Fusion proteins SAK-RGD-K2-Hir and SAK-RGD-K2-Hirul were constructed and produced in different eukaryotic expression host, *Schizosaccharomyces pombe *[17] and *Pichia pastoris* [18], respectively. Furthermore, a SAK variant (SAK-HV) with 12 amino acids thrombin-binding domain from hirudin was also constructed and expressed in *E. coli* BL21 (DE3) [19]. Two SAK variants, THR-100 and THR-174, are being developed at ThromboGenics NV [20] for the treatment of myocardial infarction for which phase III clinical trials are being conducted in India and Middle East, Africa, and other countries, respectively. The preclinical studies of these new SAK variants suggest that the novel osmotically produced multifunctional SRH protein developed in our study could be the potential candidate for such development and available in an affordable price with minimum secondary complications.

The present study was aimed to construct and express the multifunctional staphylokinase variant SRH in the osmotically inducible *E. coli* GJ1158. The biological activity of the fusion protein for its antiplatelet activity and antithrombin activity was further analyzed and compared with that of r-SAK *in vitro*.

## 2. Materials and Methods

### 2.1. Reagents

All the chemicals used in the reagents, buffers, and the preparation of media were of analytical grade and were purchased from Himedia (Mumbai, India). The restriction enzymes and the rapid ligation kit were obtained from Promega (Madison, USA) and Fermentas, Thermo Fisher Scientific Inc., Waltham, USA, respectively. PCR master mix was supplied by New England Biolabs (Schwalbach, Germany). Primers were synthesized by Sigma-Aldrich (St. Louis, MO, USA). The human plasminogen, human plasmin, human fibrinogen, fibrin, and human thrombin were also supplied by Sigma-Aldrich. The synthetic chromogenic substrates, Spectrozyme TH, and Spectrozyme PL were supplied by American Diagnostic Inc., USA. Streptokinase was purchased from Cadila Pharmaceuticals, India. Adenosine diphosphate (ADP) for the platelet aggregation assay was supplied by Chronolog Corp. (Havertown, USA). The plasmid purification kit, PCR clean up kit, DNA gel extraction kit, and Ni-NTA column were purchased from Qiagen (Hilden, Germany). *E. coli* DH5*α* (MTCC 1652) obtained from IMTECH Chandigarh was used as maintenance host for the propagation of plasmid while *E. coli* GJ1158 (Bangalore Genei, Bangalore, India) was used as expression host. Plasmid cloning vector pRSET-A was procured from Invitrogen, Carlsbad, CA, USA.

### 2.2. Cloning of Mature SAK Gene (SAK) in pRSET-A Expression Vector

The mature SAK (staphylokinase) without the N-terminal signal peptide of 27 amino acids was considered for cytosolic expression of fusion protein in the *E. coli*. Mature SAK gene (SAK) was amplified by using F: 5′-CGCGGATCCTCAAGTTCATTCGAC-3′ and R: 5′-CCGGAATTCTTTCCTTTCTATAACAAC-3′ primers from the full length native SAK gene (489 bp) that was previously isolated from *Staphylococcus aureus* in our lab [21]. The forward and reverse primers have *Bam*HI and *Eco*RI restriction sites, respectively. The amplification reaction was performed in Eppendorf Master Cycle gradient AG 22331 (Model 5331), using the program that starts at 95°C for 5 min followed by 35 cycles of three steps PCR cycle at 95°C for 30 s, 54°C for 30 s, 72°C for 30 s, and final extension at 72°C for 10 min. The polymerase chain reaction (PCR) was performed in 10 ng template in a 50 *μ*L reaction using 2 X Phusion High-Fidelity PCR Master Mix (New England Biolabs, Schwalbach, Germany) at optimum primer concentrations of 10 pico moles.

The amplified 420 bp PCR product was purified with PCR Clean-up Kit (Sigma-Aldrich, St. Louis, MO, USA) and then digested with *Bam*HI and *Eco*RI restriction enzymes at 37°C for 1 h. The resulting fragment of SAK gene was ligated into expression vector pRSET-A. The recombinants were identified with PCR and restriction enzyme analysis. Consequently, the recombinant DNA pRSET-A-SAK was further verified by DNA sequencing (Eurofins Genomics India Pvt. Ltd, India).

### 2.3. Synthesis and Cloning of RGD-Hirulog Gene

The synthetic gene coding both the antiplatelet RGD peptide (Arg-Gly-Asp) and the 20-amino-acid antithrombin Hirulog-peptide (FPRPGGGGNGDFEEIPEEYL) was designed so that it has to be linked at the C-terminal of SAK gene. These peptides were separated from each other as well as SAK by two six-amino-acid linkers (AASGGG) to retain functionality. This synthetic gene was constructed by mutual priming of a 69 mer oligonucleotide (forward: 5′-CCGGAATTCGCAGCAAGTGGCGGCGGCAGAGGCGATGCAGCAAGTGGCGG**CGGCTTTCCGAGACCGGGC**-3′) and 73 mer oligonucleotide (reverse: 5′-CCCAAGCTTCAAGTACTCCTCTGGAATCTCCTCAAAATCGCCATTGCCGCCGCC**GCCCGGTCTCGGAAAGCCG**-3′) of which 19 nucleotides are complementary at 3′end. The restriction enzyme sites *Eco*RI and *Hind*III were incorporated in the forward and reverse primers, respectively, to subsequently ligate them with SAK. The PCR amplification for mutual priming was programmed in Eppendorf Mastercycle gradient at 95°C for 2 min followed by 30 cycles of three steps cycle at 95°C for 60 s, 62°C for 30 s, 72°C for 60 s, and final extension at 72°C for 10 min. 50 *μ*L PCR reaction at 10 pico moles primer concentration using 2X Phusion High-Fidelity PCR Master Mix was performed. The amplified 123 bp PCR product was digested with *Eco*RI and *Hind*III enzymes and ligated into expression vector pRSET-A.

### 2.4. Cloning of “SAK-RGD-Hirulog” into pRSET-A Expression Vector

The final recombinant DNA “SAK-RGD-Hirulog” (SRH) was constructed by multiple ligation reactions as described by the manufacturer using T4 rapid DNA ligation kit (Fermentas, Thermo Fisher Scientific Inc.,Waltham, USA). The previously amplified and restriction-enzyme-digested PCR fragments, “SAK” (420 bp) and “RGD-Hirulog” (123 bp) mixed in 1 : 4 proportions, were added to the vector pRSET-A in 1 : 3 ratio for ligation in a 40 *μ*L reaction. The reaction mixture was incubated at 22°C for 1 h with T4 DNA ligase enzyme (5 U/*μ*L). 

The recombinant plasmid SRH “SAK-RGD-Hirulog” thus generated was transformed into chemically competent cells of *E. coli* DH5*α*. The positive transformants were selected by PCR screening and restriction enzyme digestion. The recombinant DNA, pRSET-A-SAK-RGD-Hirulog, was further confirmed by DNA sequencing (Eurofins Genomics India Pvt. Ltd, India). 

### 2.5. Expression, Production, and Purification of Fusion Proteins

The recombinant plasmids pRSET-A-SAK and pRSET-A-SAK-RGD-Hirulog were transformed into *E. coli* GJ1158 for expression study by calcium chloride method [22]. The empty pRSET-A vector was also transformed into *E. coli* GJ1158 as a control. The transformed *E. coli* GJ1158 cells were grown at 37°C for overnight in 10 mL LB medium without sodium chloride (LB/ON) supplemented with 100 *μ*g/mL of ampicillin. The overnight culture was diluted 1/50 (v/v) into fresh LB/ON medium with ampicillin and grown to an OD_600_ of 0.6 at 37°C. The culture was then induced with sodium chloride (300 mM) for protein expression. The cells were grown for 3 h and harvested by centrifugation at 4000 g for 20 min. Cell pellet was stored overnight at −20°C. One mL of uninduced culture was taken before induction as a control. The culture was then subjected to sonication with 5 s on and 10 s off cycles for 20 min on ice, and the lysate was centrifuged at 12,000 rpm for 30 min at 4°C. The soluble protein in supernatant was collected and purified by immobilized-metal affinity chromatography (IMAC) based Ni^2+^-NTA spin kit method by following the manufacturer instructions (Quigen, Hilden, Germany). The purity of the recombinant protein was analyzed on 15% sodium dodecyl sulfate-polyacrylamide gel electrophoresis (SDS-PAGE) [23], and the concentration of the purified SRH was estimated by Lowry's method [24] with bovine serum albumin (BSA) as a standard. The activity of un-induced and induced protein under different induced concentrations was tested on heated plasma agar plate as described in previous study [25].

### 2.6. Fibrinolytic Activity Assay on Fibrin Plate

Fibrinolytic activity was determined by slightly modified fibrin plate method from the previously described protocol [26, 27]. Five mL of 1% (w/v) fibrinogen solution in 50 mM sodium phosphate buffer (pH 7.4) was mixed with the equal volume of 2% (w/v) agarose solution containing 25 *μ*L of a thrombin solution (240 NIH U/mL) and 10 *μ*L of plasminogen (10 U/mL) and poured into a petri dish. The solution in the petri dish was allowed to solidify at room temperature for 1 h to form a fibrin clot layer. 20 *μ*L of various concentrations (0.1–0.8 mg/mL) of either SAK or SRH was loaded into the wells punched (5 mm) in the fibrin plate. The plate was then incubated for 6 h at 37°C. The international units (I.U.) of the enzyme activities of proteins were estimated by measuring the mean diameter of the clear zone around the well according to the standard curve plotted using the clinically standard bacterial thrombolytic agent streptokinase. 

### 2.7. Human Plasminogen Activation Assay

The plasminogen activation that determines the conversion of human plasminogen to human plasmin by either SAK or SRH was performed as in previously described method [28, 29]. 10 nM of purified SAK or SRH was added to 1 *μ*M plasminogen in 50 mM Hepes buffer (pH 7.4), with 0.01% Tween80. Consequently, a chromogenic substrate spectrozyme PL (American Diagnostic Inc., USA) was added to a final concentration of 0.3 mM. The chromogenic release of *p*-nitroanilide was measured on a microplate reader (Qualisystem, Mumbai, India) at 405 nm for 30 min at 37°C [28]. Initial activation rates were obtained from linear plots of the concentration of plasmin generated versus activation time.

The Kinetic constants *K*_*m*_, *K*_cat_ and catalytic efficiency *K*_cat_/*K*_*m*_ of plasminogen activation were derived from Lineweaver-Burk plots by a method described earlier [29, 30]. The molar concentrations of SAK and SRH were calculated by taking their molecular masses as 23 kDa and 27 kDa, respectively (estimated against standard protein marker on SDS-PAGE). An equimolar mixture of SAK/SRH and plasminogen was preincubated for 10 min at 37°C in 100 mM phosphate buffer pH 7.4, containing 25% glycerol (v/v) and then stored on ice. The preincubated activator complex (10 nM) was then incubated with different concentrations of plasminogen (0.625–10 nM final concentration) dissolved in 0.1 M phosphate buffer (pH 7.4) at 37°C [29]. The plasmin generated by the protein/plasmin complex was continuously measured for 10 min at 405 nm in the presence 0.3 mM Spectrozyme PL as substrate (American diagnostic Inc, USA). 

### 2.8. Antithrombin Activity

The antithrombin activity of SAK or SRH was determined as described earlier [18]. The assay measures the amidolytic cleavage of chromogenic substrate Spectrozyme TH (American Diagnostic Inc, USA) in presence of human thrombin. A decrease in absorbance (*A*_405_) at 405 nm in presence of SAK/SRH indicates antithrombin activity. 0.2 mM of SAK or SRH was incubated along with 2.5 *μ*M thrombin dissolved in 50 *μ*L tris-buffered saline (pH 7.4), containing 0.1% BSA for 1 h at 37°C. Subsequently, Spectrozyme TH was added at a final concentration of 0.3 mM. The thrombin activity was measured as an increase of *A*_405_ in a microtiter plate reader (Qualisystem, Mumbai, India) for 20 min at 37°C. The antithrombin activity was expressed as an inhibition of thrombin activity caused by proteins.

### 2.9. Fibrin Clot Lysis Assay

 Fibrin clot lysis assay was performed in 96-well microtiter plates as described earlier [7, 31]. Fibrin clots were formed by incubating human thrombin (1 NIH unit/mL) and human fibrinogen (1 mg/mL) in the wells of microtiter plates for 3 h at room temperature. The clots formed were later washed with HBS. 100 *μ*L freshly prepared human plasminogen (1.5 *μ*M) along with concentrations of SAK or SRH (25 nM, 50 nM, and 100 nM) in HBS was layered on each clot. After 30 min of incubation at room temperature, the surface of the clots was washed with HBS and layered with 1.5 *μ*M human plasminogen in HBS. The turbidity of the wells was measured as the A_405_ for every 10 min till the turbidity reached the minimal value. The time to achieve 50% lysis of clot (*T*_50%_) was calculated from the OD/time data. Similarly, concentration required to obtain 50% clot lysis (C_50_) was also determined from clot lysis (%) versus protein concentration at a fixed time of 3 h. The experiment was repeated thrice with HBS as negative control.

### 2.10. Platelet Rich Clot Lysis Assay

Platelet rich plasma (PRP) was prepared by a 2-step centrifugation of whole blood [32]. The whole blood was aliquoted into tubes containing anticoagulant 3.2% trisodium citrate (9 : 1). The blood was centrifuged at 200 g for 15 min to separate red blood cells (RBCs) from platelets and plasma. The supernatant platelets and plasma were further centrifuged in sterile conditions at 400 g for 15 min to pellet the platelets. The platelets were later resuspended in plasma to achieve platelet concentration 4-5 folds above the physiologic levels.

Platelet rich clots were prepared by mixing human thrombin (1 NIH unit/mL), human fibrinogen (1 mg/mL), and 20 mM CaCl_2_ to human platelet rich plasma at room temperature [33]. 100 *μ*L of polymerizing solution per each well was incubated at room temperature for 3 h in microtiter plate to form clots. The clots were washed with HBS and then layered with 100 *μ*L fresh aliquot of 1.5 *μ*M human plasminogen and various concentrations of SAK or SRH (25, 50 and 100 nM). After 30 min each clot was washed with HBS to remove unbound protein and later flooded with 100 *μ*L of 1.5 *μ*M human plasminogen. The clot lysis process was monitored by measuring the turbidity in a microtiter plate reader. *T*_50%_ and C_50_ for clot lysis for the proteins were calculated from absorbance against time. The experiment was repeated thrice with HBS as negative control.

### 2.11. Antiplatelet Aggregation Activity Analysis

The platelet aggregation was measured by whole blood aggregometer (Model 591/592, Chrono-Log Corp., Havertown, PA, USA) using an electrical impedance method [34]. Briefly, 500 *μ*L of citrated human blood (9 : 1 of blood to 3.8% sodium citrate solution, v/v) was diluted with equal volume of 0.9% NaCl in plastic cuvette and constantly stirred in aggregometer at 200 g and 37°C. The electrode with two fine palladium wires was inserted in the sample cuvette. The platelets in the blood sample adhere to palladium wires, forming uniform monolayer of platelets coating the wires. A small voltage difference was applied across the two wires, and the impedance caused by the platelets coating the wires was measured. The impedance in untreated sample with no aggregation is constant and produces a baseline. In presence of agonist, more platelets accumulate and increase the impedance between the wires. The increase in impedance is measured in ohms (Ω). 10 *μ*L of the SAK or SRH at various concentrations (5 *μ*M, 10 *μ*M, 15 *μ*M, 20 *μ*M, and 25 *μ*M) was added to the dilute whole blood and incubated for 5 min at 37°C before addition of agonist ADP (10 *μ*M). Aggregation curves were recorded for 6 min and analyzed using AGGROLINK software. The percentage inhibition of platelet aggregation was calculated as follows:

(1) $$\begin{array}{c} \%\;\;\text{Inhibition}=\left(1-\frac{\text{Aggregation}\;\text{of}\;\text{sample}}{\text{Aggregation}\;\text{of}\;\text{control}}\right)\times 100 \end{array}$$

### 2.12. Statistics

 All the values presented in figures and tables are the mean ± standard deviation (SD) of 3 independent experiments. Student's *t* test or one way analysis of variance (ANOVA) was used where applicable. Probabilities of less than 5% (*P* < 0.05) were considered statistically significant.

## 3. Results and Discussion

### 3.1. Synthesis of SRH Recombinant Vector

**Figure 1:**
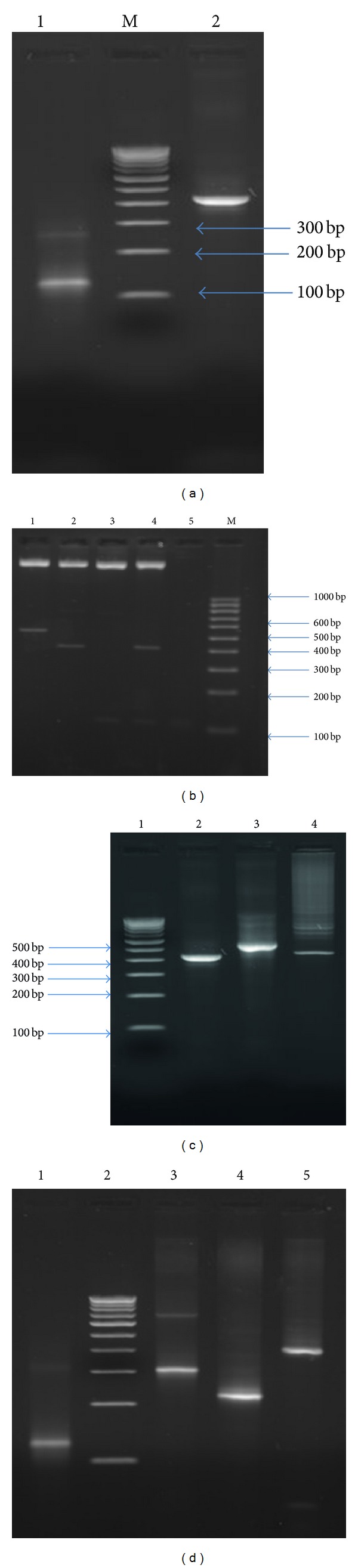
Cloning of “SAK- RGD- Hirulog” (SRH) and SAK gene into pRSET-A. (a) Amplification of mature SAK and synthetic gene, lane 1: amplification of mature SAK of 420 bp; lane 2 : 100 bp DNA ladder; lane 3: Mutual amplification of synthetic gene of 123 bp. (b) Restriction digestion analyses of SRH, mature SAK and RGD-Hirulog, Lane 1: Double digestion of recombinant plasmid pRSET-A-SRH. The SRH gene of size 543 bp was pop out; Lane 2: Double digestion of recombinant plasmid pRSET-A-SAK. The SAK gene of size 420 bp was pop out; Lane 3: Double digestion of recombinant plasmid pRSET-A-RGD-Hirulog. Synthetc gene of 123 bp was pop out; Lane 4: Triple digestion of recombinant plasmid pRSET-A-SRH. The SAK (420 bp) and synthetic gene “RGD-Hirulog (123 bp)” were pop out; Lane 5: Synthetic gene “RGD-Hirulog” of 123 bp; Lane 6: 100 bp DNA ladder. (c) PCR confirmation of pRSET-A-SAK. Lane 1: 100 bp DNA ladder; Lane 2: Amplification of mature SAK with gene specific primers; Lane 3: Amplification of mSAK with vector specific forward primers T7U and gene specific reverse primers; Lane 4: amplification of mature SAK with gene specific forward primers and vector specific reverse primes T7T. (d) PCR confirmation of pRSET-A-RGD-Hirulog. Lane 1: Mutual amplification of synthetic gene RGD-Hirulog; Lane 2: 100 bp DNA ladder; Lane 3: Amplification of RGD-Hirulog gene with vector specific forward primers T7U and gene specific reverse primers; Lane 4: Amplification of RGD-Hirulog gene with gene specific forward primer and vector specific reverse primer T7.T; Lane 5: Amplification of RGD-Hirulog gene with vector specific primers T7U and T7U.

The SAK gene was amplified from 489 bp full length gene of SAK that was previously isolated from lysogenic *S. aureus* in our lab [21]. A single band of about 420 bp was observed on 1.5% agarose gel (Figure 1(a), Lane 1). Similarly, the synthetic gene fragment synthesized by mutual priming of oligonucleotides was observed as 123 bp fragment on 1.5% agarose gel electrophoresis (Figure 1(a), Lane 3). The amplified gene fragments of both SAK and synthetic gene were ligated into pRSET-A vector. The incorporation of the gene into the vectors was confirmed by PCR amplification of the gene fragments from the recombinant plasmid templates with vector specific forward and reverse primer against respective gene forward or reverse primers (Figures 1(c) and 1(d)).

**Figure 2:**
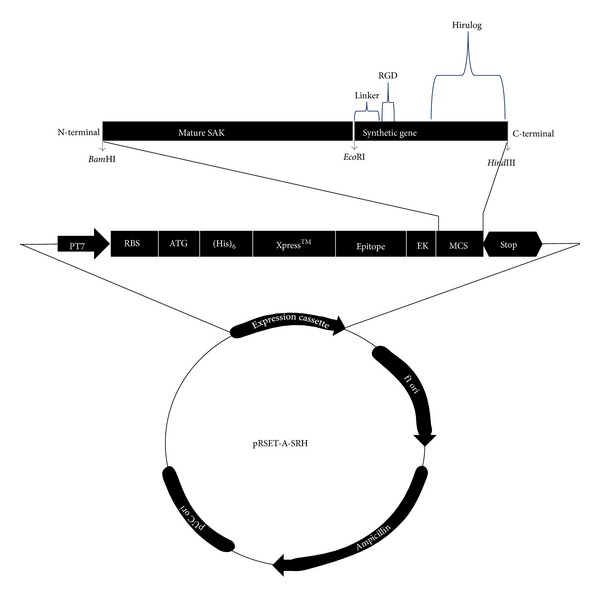
Outline of the pRSET-A-SRH plasmid (not to scale) indicating the location of SRH sequence insert. The amplified fragment of SAK and synthetic gene were inserted into the *Bam*HI and *Hind*III sites of pRSET-A expression vector by 3-way ligation reaction for the construction of plasmid pRSET-A-SRH.

Consequently, the respective gene products popped out by the restriction digestion of individual vectors were sequentially ligated together as a single gene and finally into pRSET-A to form chimeric gene SRH as pRSET-A-SRH (Figure 2). The restriction digestion analysis with respective restriction enzymes confirmed the presence of the entire gene fragments in the respective vectors that were used for further analysis (Figure 1(b)). The incorporation of the complete SRH into the pRSET-A was confirmed by triple digestion analysis of the SRH vector (Figure 1(b)). Ultimately, the recombinant vectors with SAK and SRH were further confirmed by DNA sequencing for presence of gene in the vectors. Thus the multifunctional fusion gene SRH was cloned downstream of the T7 promoter and introduced into *E. coli* GJ1158 host strain for over expression by sodium chloride.

### 3.2. Expression, Purification, and Activity Analysis of Recombinant Protein SRH

**Figure 3:**
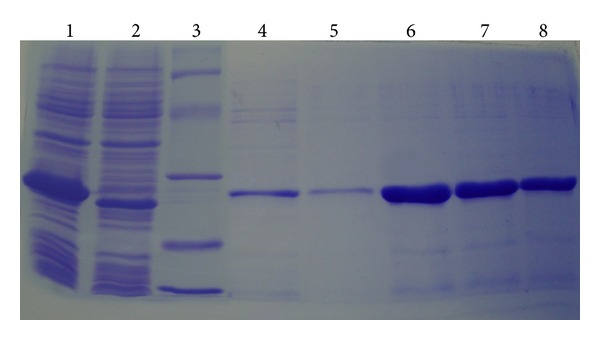
Expression and purification of SRH from *E. coli* GJ1158. Expression and purification analysis SRH under different concentrations of sodium chloride on 15% SDS-PAGE stained with coomassie brilliant blue R-250. Lane 1: SRH induced with 500 mM sodium chloride; Lane 2: SRH induced with 300 mM sodium chloride; Lane 3: Protein marker (14.3, 20.1, 29, 43, 66 97.4 kDa); Lane 4: Ist wash; Lane 5: IInd wash; Lane 4, 5, and 6: purified protein eluates.

The recombinant SRH was expressed in *E. coli* GJ1158 by sodium chloride induction and purified from bacterial cell lysates using immobilized-metal affinity chromatography (IMAC). The SDS-PAGE (15%) of cell lysate induced with sodium chloride showed an over expressed prominent protein band of molecular weight 27 kDa, approximately (Figure 3). After IMAC purification, a prominent single protein band was detected on 15% SDS-PAGE. The molecular weight of the recombinant SRH on SDS-PAGE was in reasonable agreement with molecular weight of amino acid sequence calculated theoretically. The cytosolic expression of soluble fusion protein was also optimized at two different inducing concentrations of sodium chloride (300 mM and 500 mM) and tested for activity on heated plasma agar plate. The highest expression of SRH was at 300 mM compared to 500 mM evident from a more prominent band on SDS-PAGE which may be due to deleterious effect of high salt concentration on the cells growth. The activity of the purified proteins was also confirmed on heated plasma agar plate. 

The multifunctional fusion protein (SAK-RGD-Hirulog) was expressed in GJ1158 as soluble form with a yield of 0.27 g/L which is more than the 55% of total protein. The yield is comparable to the values reported for the similar type of construct derived from the *E. coli* BL21 (DE3) as well as yeast [17–19]. 

### 3.3. Fibrinolytic Activity of Recombinant SRH

**Figure 4:**
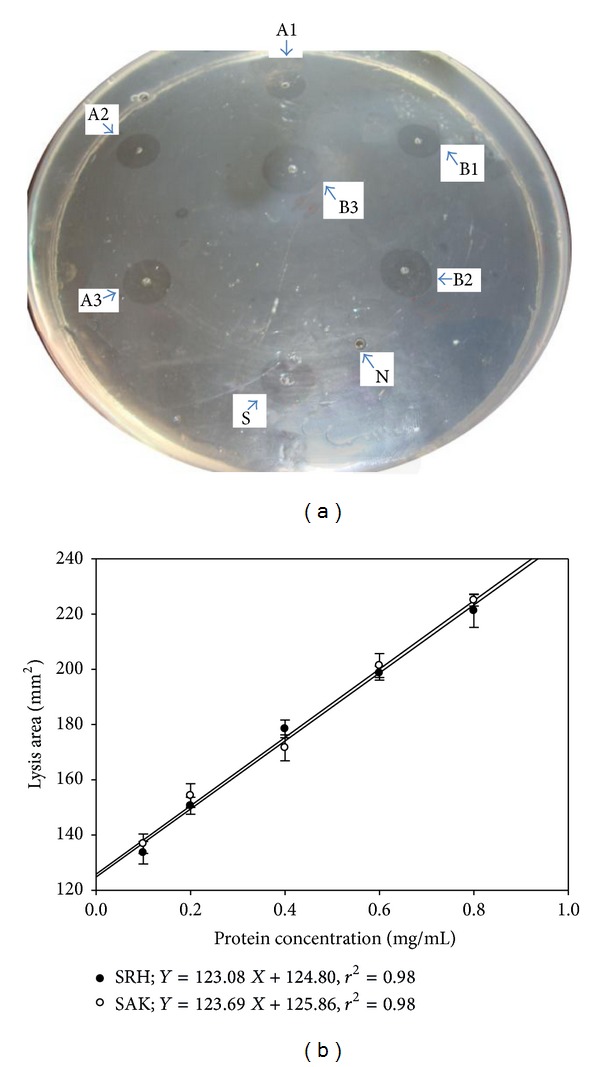
Comparison of fibrinolytic activity of SRH and SAK. The fibrinolytic activity was monitored by incubating the fibrin plate containing different concentrations of SRH or SAK at 37°C for 6 h. (a) Results are representative of three independent experiments. The wells A1, A2, and A3 loaded with recombinant SAK while B1, B2, and B3 contain SRH. The well S represents the positive control with 2000 IU of streptokinase while N as a negative control with HBS. 20 *μ*L samples of different concentrations (0.1, 0.2, and 0.4 mg/mL) were loaded into the wells of fibrin plate. The lytic areas are shown as clear zone. (b) Fibrinolytic regression curve of SRH and SAK. The areas of clear zones were plotted over the various concentration proteins. The data shown are mean of three independent experiments.

Fibrinolytic activities of SRH and SAK were measured against the standard streptokinase on fibrin plate using clearance zone method. The fibrinolytic activities of SAK and SRH (Figures 4(a) and 4(b)) were measured as 102, 955 IU/mg, and 102, 730 IU/mg respectively. There was no significant difference in the activities of SAK and SRH. The results revealed the functionally active fusion of SAK protein with RGD peptide and Hirulog without any significant alteration in biological properties of fusion protein. 

### 3.4. Activation of Human Plasminogen to Plasmin by SRH

**Figure 5:**
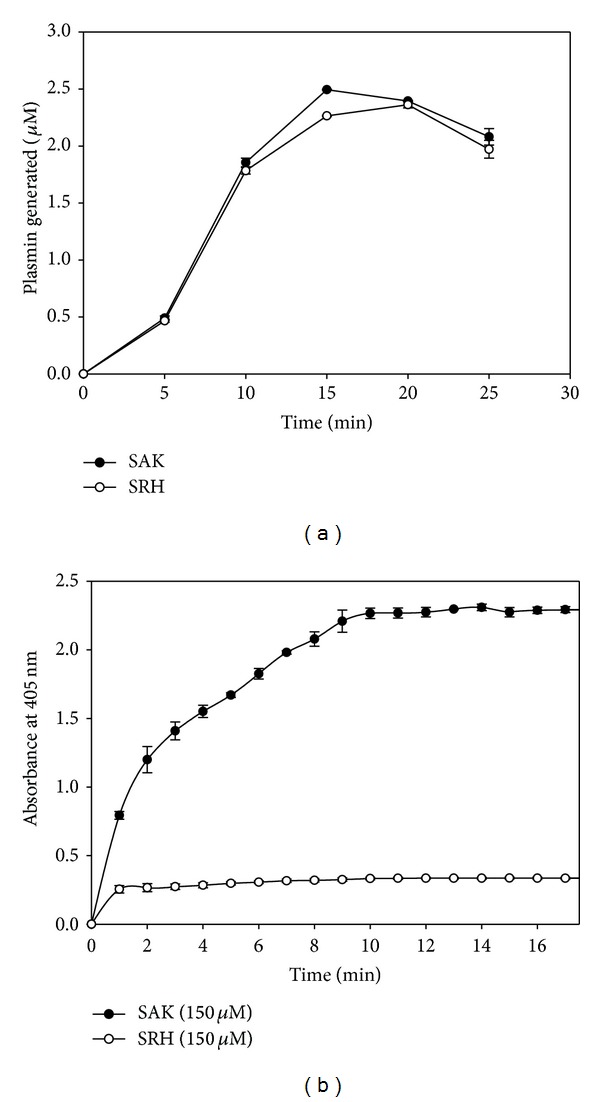
(a) Time course activation of plasminogen by SRH and SAK. The plasminogen activation activities of SRH and SAK were determined as a function of generated plasmin. Plasminogen (5 *μ*M) and SRH or SAK (10 nM) were incubated at 37°C. At different time point, aliquots were withdrawn and assayed for plasmin activity with Spectrozyme PL (0.3 mM). Data represent a mean ± S.D. of three independent experiments performed under same condition. (b) Antihrombin activity of recombinant protein SRH and SAK. The Antithrombin activity was expressed as inhibition of amidolytic activity of thrombin. Thrombin (200 nM) activity was estimated with Spectrozyme TH (0.3 mM) at the final concentration of 150 *μ*M of SRH or SAK. Data represent a mean ± S.D. of three independent experiments performed under same condition. There are significant statistical differences of antithrombin activity between SRH and SAK (*P* < 0.05).

Plasminogen activation was determined by plasminogen-coupled chromogenic substrate assay. It was observed that SRH had almost similar plasminogen activation profile as SAK. The plasminogen activation reached a steady state plateau after 15 min and about 78% of plasminogen was activated to plasmin after 10 min incubation with enzyme (Figure 5(a)). There was an initial lag phase of 2 min before the enzyme activatation. This is because the catalysis of plasminogen to plasmin needs a preformed plasmin and staphylokinase complex to actually catalyze the formation of plasmin [31]. 

The kinetics of plasminogen activation by preformed complexes SRH or SAK with plasmin were also compared. The overall enzyme catalysis obeyed Michaelis-Menten kinetics, and the Michaelis constant (*K*_*m*_), catalytic rate constant *K*_cat_, and catalytic efficiency *K*_cat_/*K*_*m*_ of SRH were similar to SAK. A comparison of the kinetic parameters (Table 1) shows SRH has slightly higher values than SAK however not statistically significant. All these results revealed that mature staphylokinase interacts with plasmin in a similar manner either it is present alone or as a part of fusion protein and forms a binary complex with plasmin in 1 : 1 ratio with equivalent plasminogen activating potential. The SRH has similar kinetic properties to SAK despite being modified for antiplatelet and antithrombin activities.

### 3.5. SRH Exhibits Antithrombin Activity

The antithrombin activity was measured as inhibition in amidolytic cleavage of chromogenic substrate caused by thrombin in the presence of either SAK or SRH. Hirulog, a 20-amino-acid (FPRPGGGGNGDFEEIPEEYL) antithrombotic hirudin analog which has shown efficacy in several models of thrombosis by increasing the reperfusion rate or decreasing the incidence of reocclusion [35], was used in the construction of multifunctional staphylokinase variant. The recombinant SRH at 150 *μ*M significantly (*P* < 0.05) inhibited the thrombin activity after 1 min compared to SAK (Figure 5(b)). This strong inhibition by SRH infers that the Hirulog peptide fragments in SRH are accessible to thrombin and thus inhibit thrombin catalytic activity. 

### 3.6. Fibrin Clot Lysis Potential by SRH Is Similar to SAK

**Figure 6:**
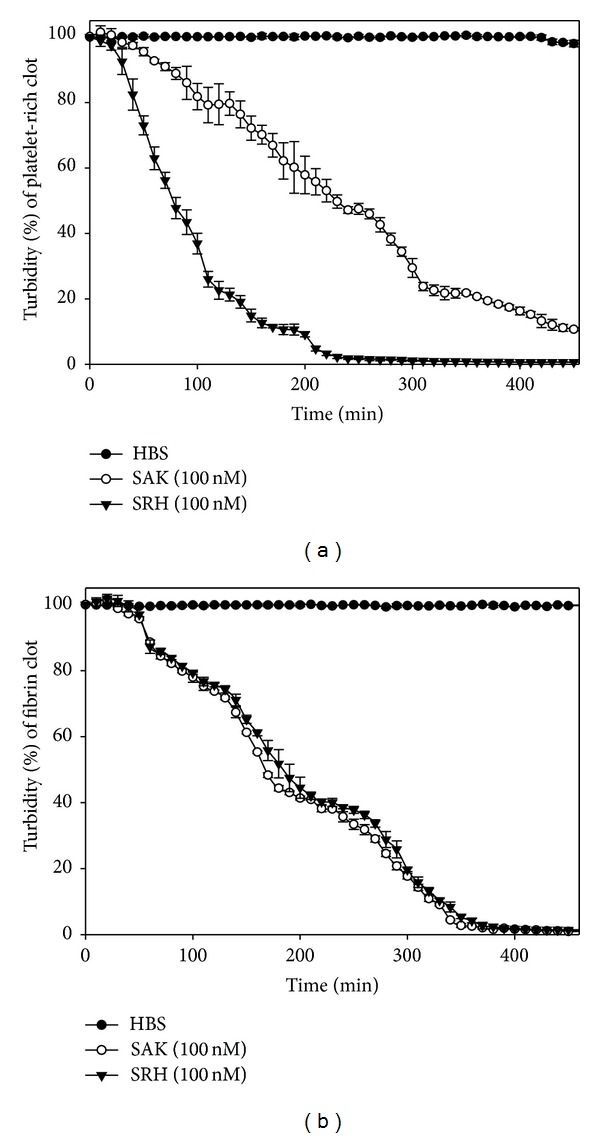
Time course of turbidimetric fibrin clot and platelet-rich clot lysis assay. Fibrin clot or platelet-rich clots were incubated with HBS or with recombinant protein SRH or SAK (100 nM) in a solution containing 1.5 *μ*M plasminogen. The decrease in *A*_405_ with time was used to calculate the relative clot turbidity at different time point. The experiments were repeated for three times and data represent the means ± S.D. (a) Platelet-rich clot lysis assay: *T*_50%_ of SAK and *T*_90%_ of SRH were found 226.98 ± 4.62 min and 76.54 ± 4.74 min, respectively, at the final concentration of 100 nM. Control clots treated with HBS only showed that readings were stable throughout the incubation period. (b) Fibrin clot lysis assay: The *T*_50%_ of SAK (186.70 ± 2.76 min) and *T*_50%_ of SRH (191.46 ± 6.65 min) were not significantly different at the final concentration of 100 nM.

The fibrin clot lysis potential was examined by calculating the time required for 50% fibrin clot lysis (*T*_50%_). It was observed that the *T*_50%_ of the SAK and SRH protein on the fibrin clot was not significantly different at various concentrations (Figure 6(b), Table 2). The concentration of either SRH or SAK to achieve 50% lysis of clots at third hour was also calculated (C_50_). The C_50_ of SAK and the C_50_ of SRH were 93.21 ± 5.6 nM and 97.72 ± 7.5 nM, respectively, which were also not significantly different. It is thus evident from the similar *T*_50%_ and C_50_ values that the introduction of Hirulog and RGD sequence to the SAK did not affect the fibrinolytic action of SRH. 

### 3.7. SRH Is Efficient in Lysis of Platelet Rich Clots

The enhanced affinity of SRH toward the platelet for faster clot lysis was evaluated in platelet rich clot lysis assay. The time required for 50% platelet rich clot lysis (*T*_50%_) for SRH (76.54 ± 4.74 min) was significantly (*P* < 0.05) less than SAK (226.98 ± 4.62) (Figure 6(a)) at equimolar concentration. This was further demonstrated at various concentrations of SRH and SAK in Table 2 which indicates that compared to SAK, the *T*_50%_ of SRH protein was significantly (*P* < 0.05) shortened by 40%, 57%, and 63%, respectively, at their corresponding concentrations (25 nM, 50 nM, and 100 nM). Further, the C_50_ of SRH (48.71 ± 6.1 nM) was significantly (*P* < 0.05) 4-fold lower than that of SAK (187.20 ± 4.6 nM) at third hour of clot lysis and required its low concentration to obtain 50% clot lysis. Thus, these studies reported the significant enhancement of efficiency of SRH in clot lysis of platelet rich clots, and it may be concluded that SRH targets the platelet rich clot due to the direct selective binding to the activated platelet through GPIIb/IIIa receptor at the platelet rich thrombus and enhances the thrombolytic activity.

### 3.8. Antiplatelet Aggregation Assay

**Figure 7:**
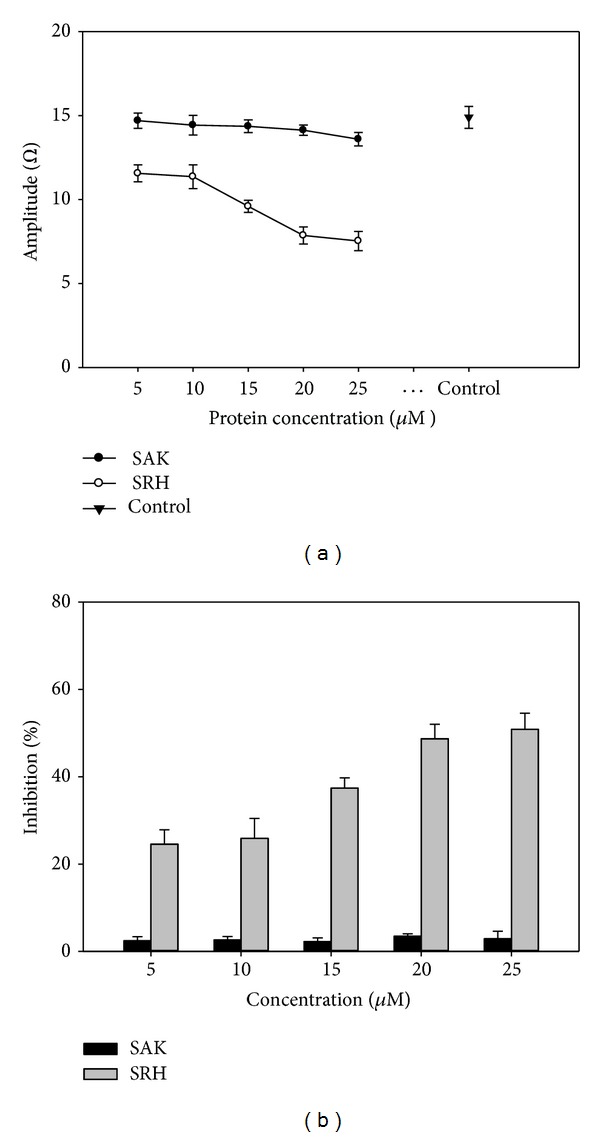
The effect of SRH and SAK on inhibition of ADP-induced platelet aggregation. (a) The amplitude-impedance aggregation results are displayed as ohms (Ω) at six minute. The SAK had negligible effect on inhibition platelet aggregation compared to SRH and did not increase with dose. Data represents a means ± SD of 3 independent experiments, which were performed in duplicate. (b) Whole blood aggregometer was used for antiplatelet aggregation study of SRH or SAK in whole blood. Data represent a means ± SD of 3 independent experiments, which were performed in duplicate. Results are expressed as a percentage inhibition in relation to platelets incubated with no recombinant proteins taken as 100%. There are significant statistical difference between the inhibitory effect of SRH and SAK for each concentration (*P* < 0.05).

Whole blood aggregometer has been used to study the ADP induced *in vitro* antiplatelet aggregation activity of SAK and SRH in whole blood instead of PRP (platelet rich plasma). This method allows the study of platelets in their physiologic milieu of whole blood like *in vivo* situations, without the centrifugation and the specimen handling required to produce platelet-rich plasma.The platelet aggregation in Figure 7(a) was shown as the change in electrical impedance (ohms (Ω)). The percentage of inhibition in platelet aggregation by SAK or SRH at various concentrations was calculated with respect to untreated sample (100%). The SAK had negligible effect on inhibition of platelet aggregation at all doses. In contrast, SRH showed significant inhibition of platelet aggregation compared to SAK (*P* < 0.05). The SRH showed significant dose-dependent inhibition of platelet aggregation (Figure 7(b)). An increase in dose of SRH at doses of 5 *μ*M, 10 *μ*M, 15 *μ*M, 20 *μ*M, and 25 *μ*M showed 24.55 ± 3.28%, 25.85 ± 4.6%, 35.73 ± 2.05%, 48.69 ± 3.35%, and 50.85 ± 3.71% inhibition, respectively. The results of this study revealed that addition of RGD peptide as platelet targeted domain to the mature staphylokinase led to the accomplishment of the ability to inhibit the platelet aggregation with significant value. However, at higher doses (20 *μ*M and 25 *μ*M), the increase in percentage of inhibition was comparatively low which infers that the receptor GPIIb/IIIa on activated platelet was sufficiently blocked by SRH even at its low concentration and saturated when the dose was increased. 

## 4. Conclusion

The present study demonstrates that the fusion protein is more potent and is a faster thrombolytic agent with antithrombin and antiplatelet activities compared to native SAK. The fusion protein was expressed and purified successfully from the osmotic inducible *E. coli* GJ1158 without the loss of any of its functional properties, and the yield is comparable to the production from the *E. coli* BL21 (DE3) and yeast. *In vitro *results revealed that multifunctional fusion protein along with its fibrinolytic activity effectively inhibits the thrombin activity and platelet aggregation. To verify the therapeutic potential of multifunctional protein SRH *in vivo*, the animal model of thrombosis was planned which would be worthy for clinical trial. The multifunctional fusion protein produced in the present study can be developed into an efficient candidate that will be able to replace the existing drugs with reduced reocclusion problem.

## Figures and Tables

**Table 1:** Kinetic parameters of equimolar complexes of plasmin with SAK and SRH.

Protein molecule	*K*_*m*_ (*µ*M)^a^	*K*_cat_ (S^−1^)^a^	*K*_cat_/*K*_*m*_ (*µ*M·S)^−1^ ^a^
SAK	2.58 ± 0.24	1.62 ± 0.07	0.058 ± 0.03
SRH	2.64 ± 0.26	1.63 ± 0.04	0.059 ± 0.02

^a^The results are mean ± S.D of three independent experiments.

**Table 2:** Summary of *T*_50%_ of SAK and SRH.

Protein	Concentration (*µ*M)	*T*_50%_ of Fibrin clot (min)^a^	*T*_50%_ of platelet rich clot (min)^a^
SAK	25	221.66 ± 8.27	249.83 ± 3.90
SAK	50	209.29 ± 5.95	234.72 ± 7.17
SAK	100	186.70 ± 2.76	226.98 ± 4.62
SRH	25	218.58 ± 7.81	133 ± 5.37
SRH	50	213.63 ± 8.15	91.34 ± 6.98
SRH	100	191.46 ± 6.65	76.54 ± 4.74

^a^The results are mean ± SD of three independent experiments.
